# Mr. Cooke, on Erysipelas

**Published:** 1804-12-01

**Authors:** Robert Cooke

**Affiliations:** London


### *To the Editors of the Medical and Phyſical Journal.*

GENTLEMEN,

I Was much pleased the other day by the perusal of an observation made by Mr. Cuming, on the treatment of erysipelas, in your last valuable Journal. On account of

 its its according so exactly with the plan I have been in the habit of seeing pursued, and myself adopting to a very considerable extent for the last five years; I allude to the practice of applying cold lotions in this disease, and to those more particularly which appear about the head and face, where we ought, as soon as possible, to check the complaint, particularly in females, in consequence of its so frequently disfiguring them. Mr. Cuming very justly observes, the celebrated Cullen conceived the practice to be injudicious, and recommended the principal reliance to be placed on flour; but from what I have seen of the disease within the time before mentioned, and which has not been merely a few solitary cases now and then, I am confident that this plan is by no means the best; for I do not recollect one case where the application of cold lotions were early and properly applied, but it prevented suppuration and all its ill consequences, not only in the head and face, but the extremities; in the former I have used it very freely and repeatedly, when the inflammation has run extremely high, both locally and constitutionally, and I never once found by repelling it that either phrenitis, peripneumony, angina, or any unpleasant complaint followed; indeed, to me, the propriety of their application appears very obvious, upon the simple principle of checking that inflammation, which, if allowed to go on, would probably extend to the brain, or some other important part, and in this way prove fatal. The lotion which has been used so extensively in the practice I have had an opportunity of attending to, has been the aqua aluminis composta (P. L.) and which I think is preferable to the cerussa acetata, on account of its being a better tonic; the other I conceive to be more applicable to those kind of inflammations which more particularly require sedatives.

In a case which occurred about twelve months back, and which was particularly bad, the face being so much disfigured by the swelling as for the man's friends not to know him; he, from the first of my visiting him, used cold applications to the part; his bowels were kept open, and saline medicines administered, as the febrile symptoms seem to indicate, and he got perfectly well. This man would not bear bleeding: indeed, I have generally found, that if phlebotomy is performed in the early stage of this disease, although the inflammatory symptoms run pretty high, the patients seldom do so well as if it had been omitted; for, after it, I have almost constantly found the pulse pulse has gradually sunk, and the part put on that degree of sluggishness and livid hue, as to require a very different mode of treatment to that adopted at first; but I dont mean to say, that because this is the case very frequently in a large town like this, that it should be so in the country, where I believe venæsectio very generally becomes necessary in the treatment of this disease, and for two very obvious reasons. First, the lower class of people here (and amongst whom my practice is principally confined) live chiefly on gin, porter, and tea, and take very sparingly of animal food; and, secondly, their living in a large city where the air cannot possibly be so pure as if they resided many miles from it. This then I think will very satisfactorily explain why, in this respect, the plan of treatment here and that in the country may very properly, somewhat vary. It is a very common circumstance to find that erysipelas in the face and other parts, if considerable, will leave an œdema with a scurfiness, which was the case in the man whose case I have just mentioned; but it was entirely taken away, by giving him a grain of calomel every night, and the cortex in the day. This practice is generally adopted under such circumstances, and commonly proves amply sufficient.

Surely then, if a disease so very troublesome and indeed formidable, as erysipelas, can be checked, and suppuration prevented by this mode of treatment, and that without any risque to the person's general health, it is a very cogent reason for adopting it. We are told by that ancient writer Hippocrates, that a suppuration and sloughing of this kind, is of a very serious nature; yet he was by no means an advocate for repellants under such circumstances, for in his Aphorisms, he says, if there is a metastasis of the morbific matter from within, outward, the habit will be relieved, and the patient recover; but the sudden return of a humour inward is a bad sign, and imagined some formidable disease would be brought on in consequence. But their constant application proves this is not the case, also the advantages derived by their use to be very great, and the danger merely imaginary, that I hope, ere long, they will be universally employed; and I am sure no practitioner, who is anxious for his patient's speedy recovery, (and which, I have no doubt, is the case with every one) would, after employing it once, ever again, under similar circumstances, omit using it a second and indeed for ever after; for, as I have before stated, it prevents, if early employed, any serious mischief coming on. Whereas by sprinkling sprinkling the part with flour, using soft emollient fomentations and cataplasms, &c. we very often find the inflammation continues to remain, the part suppurates and sloughs, and instead of curing your patient in a few days, or at farthest in a week or fortnight, he is confined for many weeks or perhaps several months.

I am well aware that many, and indeed most, modern practitioners will object to the treatment of this disease in the way I have mentioned, and will say, it is obvious that every thing which can occasion a retrocession of the morbific matter from the surface of the body must prove injurious, and therefore they contend every erysipelas should be encouraged, for that, in proportion as the external inflammation advances, does the fever subside. This argument may do extremely well to serve the purpose of the theorist, when discussing the subject in a medical society; but if he will turn his attention only a little to the practical part of his profession, and observe minutely and impartially the effects of the plan proposed, I am sure he will soon be convinced of the false grounds upon which his arguments are established.

Sometimes, and indeed not very unfrequently, in consequence of the inflammation being very great at first, and the part entirely neglected, suppuration will come on, which generally is of a phagadenic and gangrenous kind, (very seldom proving favourable) the best application, under such circumstances, seems to be the strong beer grounds and oatmeal, with bark, opium, and wine internally; and for those whose circumstances will not allow them to get the latter, porter will be found no contemptible substitute; during the separation, the sore may be sprinkled with a powder composed of equal parts of the pulv. gum. myrrh, and lap. calaminaris; or instead of the latter, the cinchona may be employed, and over this the poultice is to be laid. All greasy applications are to be avoided, more particularly in the early stage of the disease, for they will invariably be found prejudicial. When the part has become perfectly healthy, the poultice is to be discontinued; but frequently the use of the powder may be persevered in with considerable advantage, and over it dry lint and a common pledget, and commonly this will be all that is necessary to complete the cure.

I embrace this opportunity of mentioning the good effects I have seen, from giving the zincum vitriolatum in the cure of intermittents, and which medicine I was induced to employ from the recommendation of the gentleman man whose name I have before mentioned. I have only had an opportunity of giving it in two cases, the first of which was a remarkable obstinate one, and had bid defiance both to the cortex and solutio arsenici; indeed, the subject was a very unfavourable one, for he had repeatedly laboured under the same disease while abroad as a soldier; he had also been a hard drinker of spirits, by which his constitution had been much shattered. Under these unfavourable circumstances, I began with the above medicine, and persevered in its use in the manner directed by Mr. C. and I am happy in saying the man had not one paroxysm after its first administration. The other case was of a much milder type, and the subject by far more favourable in every respect. In this man, I began with it from the first, and it answered my most sanguine expectations; indeed, I have much pleasure in acknowledging that I always feel great satisfaction, and receive considerable improvement, by the perusal of the above Gentleman's very valuable practical observations from time to time, and which, I trust, he will afford me frequent opportunities of repeating,

I am, &c.

*London, Nov.* 12, 1804. ROBERT COOKE.

